# Errors in handling and manufacturing of orthopaedic implants: the tip of the iceberg of an unrecognized system problem?

**DOI:** 10.1186/1754-9493-1-5

**Published:** 2007-12-05

**Authors:** Johannes K Fakler, Yohan Robinson, Christoph E Heyde, Thilo John

**Affiliations:** 1 Department of Trauma and Reconstructive Surgery, Charité University Medical School, Campus Benjamin Franklin, Hindenburgdamm 30, 12200 Berlin, Germany

## 

Patient safety related to orthopaedic implants, particularly joint arthroplasty, is a major concern due to the increasing elderly population and the wide variety of available devices. The safe use of medical devices, such as orthopaedic implants, is regulated by institutions or governmental agencies in many countries. Nevertheless, adverse incidents associated with the implantation of orthopaedic devices in patients are reported periodically in the news media.

Recently, a series of 47 consecutive patients with falsely implanted total knee arthroplasties was reported in German newspapers [1-5]. Between May 2006 and March 2007, orthopaedic surgeons in a German hospital erroneously implanted femoral components for total knee replacements in a non-cemented fashion, although these specific implants had been designed for cemented use only [1-5]. Apparently, multiple contributing factors accounted for these adverse events in which patients were harmed by a combination of human failure and system errors. Until present, 30 patients underwent a surgical revision, four patients have been scheduled for revision surgery, and the remaining 13 patients have not reported any complaints [6].

The disputed US-manufactured knee prosthesis is available in two different versions, for use with or without cement. This specific implant was introduced at the reported hospital in May 2006. A root cause analysis revealed that one of the contributing factors leading to wrong use of this implant was related to the original package labeled in English language. The labeling of the femoral component packaging as "non-modular cemented" was erroneously translated to "non-cemented" or "without cement" by the responsible hospital staff [1-4]. With respect to the German law on medical products, all devices must be delivered with an according German instruction [7]. In the present case, the US manufacturer of the knee prosthesis included a German instruction inside the package [2]. About one year later, a sticker with the German translation of "without cement" was added to the package labeling of the "true" cementless version. At the time, the responsible hospital staff suddenly realized that some femoral components designed for cemented use had been erroneously implanted in a cementless fashion [3].

Aside from this system error related to wrong language translation and interpretation, another contributing factor which led to the wrong use of this orthopaedic device might have been a misinformed representative by the manufacturing company who was present in the operating room at the time of surgery. According to the hospital's spokesperson, the components for cemented use only were sorted into the shelves designated for cementless components despite continuing supervision by the company's representative. Apparently, this particular representative attended knee arthroplasty procedures at this hospital and assisted the operative surgeons in technical issues related to this newly introduced implant [1,3].

According to the US manufacturer, no serious adverse events or technical problems have been reported within 15 years of distribution in Germany. No comments were made related to the potential aspect of an individual human failure by the company's representative [1,3].

The shocking fact that 47 consecutive patients suffered from the same basic error implies that not only human failure, but also system errors and lacking control mechanisms may have contributed to this series of adverse events.

As outlined by this alarming example, most adverse events in medical or surgical procedures are typically of multifactorial origin. This includes organisational and structural mismanagement which may be accentuated by unpredictable individual human failure [10]. A root cause analysis of the above-mentioned case scenario should help to improve patient safety related to the improper handling of orthopaedic implants in the future.

Analogous to the Food and Drug Administration (FDA) in the USA, the Federal Institute for Drugs and Medical Devices in Germany regulates the use of any medical device that is intended for use in the diagnosis, mitigation, treatment, or prevention of disease [7,11]. The FDA regulations ascertain that all labeling of medical products must be in English language [12]. The terms "label" and "labeling" are specifically defined as "display of written, printed, or graphic matter upon the immediate container of any article..." and "all labels and other written, printed, or graphic matter upon any article or any of its containers or wrappers..." [12]. The German law regulating the use of medical devices demands that product information, particularly safety issues, are provided in German language. However, this regulation does not specifically demand that labeling of packaging of medical devices must be in German language [7]. As outlined in the present case, the English language package labeling of an arthroplasty implant was the basis of a wrong translation/interpretation of the label and thus led to an improper implantation of total knees. From a human failure perspective, the distinct coating properties between the cemented and uncemented version of the femoral component (porous vs. smooth surface) were neither recognized by the instrumenting personnel, nor by the responsible surgeon at the time of surgery [1,3,4].

Another aspect to be addressed is the role of the presence of a health care industry representative (HCIR) in the operating room. According to the American College of Surgeons (ACS), industry representatives in clinical settings are assigned an important role in patient safety and quality of care by providing detailed information about the proper use of medical devices [13]. However, the surgeon in charge – as the "captain of the ship" – remains ultimately responsible for the adequate performance of surgical procedures [13,14]. On the other hand, companies manufacturing medical devices must take adequate steps to ensure a proper education and instruction of their representatives. This was obviously not warranted in the present case. With respect to the protection of patients' rights and privacy, we also question why patients' informed consent is not required for the presence of third parties in the operating room, such as HCIRs.

The establishment of regional, national, or international reporting systems and quality control registries may help avoiding repeated identical error patterns, e.g. by early detection of a discrepancy between type of implant and the implanting procedure. The Swedish Hip Registry has been established for many years now and directly influenced the number and types of available hip implants by eliminating those with poor performance [15]. Although these consequences result from long-term data, the Norwegian hip registry successfully demonstrated detection of poor cement quality and subsequent recall at an early stage [16].

Since the introduction of the Swedish hip registry in 1979, revision rates for hip arthroplasty have been reduced approximately by half, to currently 7% [15,17,18]. In contrast, the mean revision rate for total hip arthroplasty in the USA is still as high as 18% [18,19]. It is conceivable that formal registries on joint arthroplasties may help identifying implantation-related errors at an early stage and thus prevent further patients from undergoing inadequate surgical procedures.

We wish to emphasize that the particular incidence analyzed here, based on a rare event which occurred in a single German hospital, but may reflect the "tip of the iceberg" of an unrecognized risk for patient safety in joint arthroplasty. This notion is exemplified by the world-wide recall of the Interop acetabular cup by Sulzer Orthopedics (Austin, TX) due to a manufacturing error in 2001. This error was attributed to a modified cleaning process, which left oily residues on the implant's porous coating [8]. During the time of introduction of this acetabular cup in 1997 and its worldwide recall in 2001, the device had been implanted in about 17,500 patients. By 2005, about 2,500 patients had undergone revision surgeries due to the malfabricated cup [9]. A retrospective study reported a failure rate of 30% for the Interop shell within two to 17 months after total hip arthroplasty [9].

The high number of adversely affected patients in this example demonstrates that the identification and correction of manufacturing errors in joint implants may take several months to years. Obviously, in times of a global market with worldwide product distribution, any orthopaedic device may be implanted in hundreds or thousands of patients within a very short period of time. This example emphasizes the need for a systematic approach for implementation of an "early warning system" in order to improve patient safety.

In summary, patient safety in the context of routinely and frequently performed surgical procedures, such as total joint replacement, needs to be improved in a systematic fashion. In the case related to wrong translation of product labels, the unequivocal labeling should be mandatory for all product containers and wrappings in the language of the country where the implant is used.

With respect to the role of HCIRs, surgical department administrators should establish specific written policies governing the presence of third parties in the operating room. The surgeon remains ultimately responsible for correct use of orthopaedic implants and must be familiar with all specific details of instrumentation and implantation techniques. In the case where a surgeon uses an implant for the first time, we recommend a prior on table demonstration and training of correct implant handling by an HCIR. Patients should furthermore be informed about the presence of HCRIs in the operating room and provide a written informed consent for allowing non-medical third parties in the operating room.

The last column in patient safety with respect to orthopaedic implants addresses public institutions which supervise medical products and patient concerns. With regard to joint arthroplasty, studies have shown that formal registries can improve long-term patient safety by reducing complication and revision rates significantly [12-15]. Such registries must be designed in a way that allows an early identification of errors and failures, in order to avoid repetitive errors which may consecutively affect a large patient population.

## Competing interests

The author(s) declare that they have no competing interests.
